# Individual and community-level factors of perinatal mortality in the high mortality regions of Ethiopia: a multilevel mixed-effect analysis

**DOI:** 10.1186/s12889-022-12695-y

**Published:** 2022-02-07

**Authors:** Desalegn Girma, Zinie Abita, Gossa Fetene, Bamlaku Birie

**Affiliations:** 1 Department of Midwifery, College of Health Science, Mizan-Tepi University, Mizan, Ethiopia; 2 Department of Reproductive Health, School of Public Health, College of Health Science, Mizan-Tepi University, Mizan, Ethiopia

**Keywords:** Ethiopia, Perinatal mortality, Regions, Multilevel analysis

## Abstract

**Background:**

Even though perinatal mortality has declined globally; it is still the major public health concern in sub-Saharan Africa countries. Ethiopia is one of the sub-Saharan countries which contribute the highest-burden of perinatal mortality with a devastating rate in some of the regions. Therefore, this study aimed to identify the determinants of perinatal mortality in the high mortality regions of Ethiopia.

**Method:**

A secondary data analysis was done using the 2016 Ethiopian Demographic and Health Survey data. The outcomes of 4120 pregnancies reaching ≥ 7 months of gestational age were considered for the analysis. A multilevel mixed logistic regression model was fitted to identify the predictors of perinatal mortality. Finally, a statistically significant association was declared at a *p*-value of ≤ 0.05.

**Result:**

The study found that birth interval < 2 years (AOR = 3.71, 95%CI:2.27, 6.07),having no antenatal care (AOR = 2.43,95%CI:1.15,5.38), initiating breastfeeding after 1 h(AOR = 4.01,95%CI:2.49,6.51), being distant from health institutions (AOR = 1.99, 95%CI: 1.24, 3.22), having previous terminated pregnancy (AOR = 4.68, 95%CI:2.76,7.86), being mothers not autonomous(AOR = 1.96, 95%CI:1.19,3.20),being no media exposure (AOR = 2.78, 95%CI:1.48,5.59),being households ≤ 4 family sizes (AOR = 4.12, 95%CI:2.19,7.79), having ≥ 6 parity (AOR = 2.48, 95%CI:1.21, 5.22) were associated with a high odds of perinatal mortality.

**Conclusion:**

The study concludes that birth interval, antenatal care, time for breastfeeding initiation, distance from health institutions, previous history of terminated pregnancy, maternal autonomy, media exposure, family size, and parity were predictors of prenatal mortality. Therefore, programmatic emphases to maternal waiting service utilization for mothers distant from health institutions and media advertising regarding the complications related to pregnancy, childbirth and on its respective direction that the mothers should follow could reduce perinatal mortality in high mortality regions of Ethiopia.

## Background

Perinatal mortality includes both terminated pregnancies after seven months (stillbirth) and early neonatal deaths in the first week of life [1]. It is an important indicator of the quality of maternal and neonatal care in the country [2].

Perinatal mortality is one of the public health concerns; globally, nearly 2 million annual stillbirths [3] and about 2.4 million newborn death [4] have been reported in 2019, with the highest-burden in low-income centuries. The risk of stillbirth is 7.6 times higher in low-income countries whereas the risk of newborn death is 9 times higher as compared to the high-income countries [3, 5].

Even though there is a global decline in stillbirth from 2.9 million in 2000 to 2.0 million in 2019 [3] and neonatal deaths from 5.0 million in 1990 to 2.4 million in 2019 [4], the perinatal mortality rate is still high in sub-sub-Saharan Africa countries which ranges from 34.7 to 42.95 per 1000 live births [6, 7]. It covers about 42 percent of the total stillbirth [3] and 41 percent of the total newborn death [5] of the globe.

The world has launched a platform “Every Newborn Action Plan (ENAP)” in 2014 to decline stillbirths and neonatal deaths to less than 10 per1,000 total births of countries by 2035 [8]. However, neonatal death and stillbirth have been reduced stagnantly in low-income countries and such countries need to accelerate the reductions of deaths to achieve the proposed targets [9].

Ethiopia is one of the developing countries in sub-Saharan Africa that have the highest fertility rate (to 4.6 children per woman) with a median age of first birth at 19.2 years [10] and has a low physician density (0. 0769 per 1,000 people) [11].

Ethiopia has been working by endorsing “Every Newborn Action Plan” to reduce the rate of perinatal mortality though it is still one of the major public health concerns in Ethiopia. In 2016, the Ethiopian Demographic and Health Survey (EDHS) reported about 33 perinatal death per 1000 birth [10]. A systematic review conducted in Ethiopia also revealed a high perinatal death (51.3 per 1000 birth) in the year between 1997 to 2019 [12]. The overall mortality rate has variation among regions and it was devastating in some of the regions in Ethiopia. According to the 2016 Ethiopian Demographic and Health Survey (EDHS) report, perinatal mortality per 1000 live births was higher in Somali, Amhara, Harari, and Tigray regions( 50, 44, 40, and 36, respectively) as compared to the national average(33 death per 1000 live birth) [10].

Perinatal mortality is known to be associated with factors such as paritry [13–15], age of mother [13, 16], maternal education [17, 18], Attending antenatal care visits(ANC) [14–16, 19], skilled birth attendant [14], previous history of perinatal mortality [14, 16, 20], low family income, birth interval [12, 19, 20], the total number of under 5 children [20], access to participation in decision making [20], conceived during teenage [21], and place of delivery [22, 23] and residency [15], and access to clean water supply [1].

Even though Ethiopia has been working to decline perinatal mortality, by ensuring equity in health, maximizing the coverage of health facilities, and improving the quality care of maternal and child health, perinatal mortality is still very high in some regions of Ethiopia (Somali, Amhara, Harari, and Tigray) [24]. Previous studies [14, 15, 22, 25–27] conducted in Ethiopia on perinatal mortality have tried to identify individual-level determinants of perinatal mortality in specific areas. However, they didn’t assess community-level factors that may influence perinatal mortality in high prevalence regions. Therefore, the primary aim of this study was to identify the individual and community level determinant factors of perinatal mortality in these high perinatal mortality regions of Ethiopia by using nationally representative data. Identifying the individual and community level factors is significant to reduce perinatal mortality in high mortality regions of Ethiopia.

## Methods

### Data source

The Ethiopian Demographic and Health Survey (EDHS 2016) data were used, which was the fourth survey conducted nationally from January 18 to June 27, 2016. The respondents in EDHS, 2016 were selected by using a two-stage stratified sampling technique. In the first stage, 645 enumeration areas (202 in urban areas and 443 in rural areas) were selected. Then in the second stage, 28 households per cluster have been selected. The detailed sampling technique was summarized in the full EDHS 2016 report [10]. A total of 4014 mothers from the high perinatal mortality regions(Somali, Amhara, Harari, and Tigray) were included in the study. Accordingly, the outcomes of 4120 pregnancies reaching ≥ 7 months of gestational age(including twin pregnancies) were analyzed to identify factors affecting perinatal mortality. Most of the variables related to pregnancy and postnatal care in EDHS data were only collected for the last birth or last pregnancy. Such that for women who had two or more pregnancies in the 5 years preceding the survey, the last pregnancy was taken. About 11.73% of the pregnancies were excluded from the study. Those have incomplete data mainly the gestational age, birth dates, and ages at deaths. The comparison of perinatal death between the Analytic sample and missing sample was checked using the chi-square test. The result revealed that there is no significant difference in perinatal death between the Analytic sample group and missing sample (3.9% vs 4.4%, x^2^ = 0.414, P = 0.255) with perinatal mortality rate 41 per 1000 vs 44 per 1000 live birth, respectively (Appendix).

## Variables of the study

### Dependent variable

The perinatal mortality rate was a dependent variable, including both stillbirths and early neonatal deaths in the five years preceding the survey divided by all births (including stillbirths) that have a pregnancy duration of seven or more months. Stillbirth is defined as pregnancy terminated after seven or more months. Early neonatal death is death within seven days (days 0–6). The outcome variables were coded as “0” for the absence of perinatal mortality and “1” for the presence of perinatal mortality.

### Independent variables

Predictors such as maternal age (years), maternal education status, paternal education status, Sex of household, working status of mothers, family size, wealth index, media exposure, age at first birth, place of delivery, preceding birth interval, women participating in making health care decisions, parity, time to breastfeeding Initiations, postnatal care, previous history of a terminated pregnancy, antenatal visit, tetanus toxoid vaccination, and Health insurance were considered as individual-level factors. And factors such as residence, distance from health facilities, water facility, and times to reach the water source were considered as community-level factors.

For the media exposure the composite variable is created from three variables; frequency of watching TV, reading a newspaper, and listening to the radio, and coded as “yes” if an individual was exposed to all or either of the three and “No” if an individual was not exposed to at least one of these. The women’s health care decision-making autonomy was assessed as the person who usually decides to obtain healthcare. Which was categorized as women participating in making health care decisions and didn’t participate in making health care decisions (decided by their husband/partner).

The water source was leveled as improved if the source of drinking water comprises one of the following: a piped household connection, public standpipe/borehole, protected dug well or spring, and/or rainwater collection. The toilet facility was leveled as improved if it has (piped into dwelling, piped to yard/plot, piped to a neighbor, and public tap/standpipe) and not improved for (no facility/bush/field, composting toilet). wealth index, In the original it was categorized into five wealth quintiles: ‘very poor’, ‘poor’, ‘middle’, ‘rich’, and ‘very rich’. For this study, we re-coded the wealth index into three categories as ‘poor’ (poor and very poor), ‘middle’, and ‘rich’ (rich and very rich) to obtain an adequate sample in each category.

### Statistical analysis

Data were extracted using SPSS version 21 software and then exported to R version 3.5.3 statistical software for further analysis. Data editing, coding, and cleaning were done. Frequencies, percentages, and the rate were used to summarize data, and tables were used to present data. In this study, a two levels hierarchy of data was considered due to the sampling techniques used in the EDHS (multi-stage stratified cluster) data. Level one units were individual pregnant mothers in households and level two units were enumeration areas (cluster). Level one (pregnant mothers in the households) was nested at the next higher level of enumeration areas (community). Therefore, the multilevel mixed logistic regression model was fitted to identify the contributing factors of perinatal mortality at each level (individual level and community level). Both bivariable and multivariable analyses were conducted. Variables that have a *P*-value of ≤ 0.25 in bivariable two-level binary logistic regression were a candidate for multivariable multilevel logistic regression analysis. Then Variables in multilevel multivariable logistic regression were declared to be statistically significant at a *P*-value of ≤ 0.05.

Four models have been developed, With an assumption of varying intercepts across communities (clusters) but fixed coefficients. The first was the null model (Model I) fitted without predictors variables. The second model (model II) was fitted for individual-level factors and conducted to examine the contribution to the variation of perinatal mortality. Whereas the third model (Model III) was adjusted for community-level factors and used to examine the contribution for variation of perinatal mortality across the cluster. Finally, the fourth model(Model IV) was developed by combining individual and community level factors. The model goodness of fit was tested using deviance information criteria (DIC)and the model with the lowest value was considered to be the best fit model. The variation inflation factor (VIF) was used to test the multicollinearity between independent variables.

## Results

### Socio-demographic characteristics and perinatal mortality rates

Out of 3322 (weighted) pregnancies, about 2806(84.5%) were from rural mothers and 2362(71.1%) were from mothers with no formal education. The overall perinatal mortality rate among the pregnancy in high mortality regions of Ethiopia was 41 per1000 births. The perinatal mortality rate was high among mothers aged ≥ 35 years (58.57 per 1000), households ≤ 4 family size (53.66 per 1000), households who had Poor wealth index (55.42 per 1000), and mothers who didn’t have media exposure (50.79 per 1000) (Table 1).

**Table 1:** Shows that the perinatal mortality among pregnancy by socio-demographic characteristics in high mortality regions, Ethiopia, 2016

Variable	Categories	Frequency (%)	weighted Frequency (%)	unweighted number of perinatal deaths (PMR [per 1,000])	weighted number of perinatal deaths (PMR [per 1,000])
Maternal age (years)	Less 20	151(3.7)	99(3)	8(57.14)	4(43.01)
	20–24	862(20.9)	606(18.2)	33(40.69)	12(20.90)
	25–29	1135(27.5)	957(28.8)	54(49.82)	40(43.80)
	30–34	858(20.8)	664(20)	26(31.82)	19(29.60)
	≥ 35	1114(27.0)	996(30)	61(58.59)	55(58.57)
Residence	Urban	760(18.4)	516(15.5)	19(25.8)	15(30.00)
	Rural	3360(81.6)	2806(84.5)	163(51.6)	115(43.23)
Maternal education status	No education	2892(70.2)	2362(71.1)	134(49.24)	105(47.06)
	Primary	872(21.2)	700(21.1)	36(43.53)	21(31.00)
	Secondary and above	356(8.6)	260(7.8)	12(34.78)	4(16.00)
Sex of house hold	Male	3288(79.8)	2841(85.5)	153(49.27)	107(39.54)
	Female	832(20.2)	481(14.5)	29(36.80)	23(50.77)
Paternal education status	No education	2257(54.8)	2027(61.0)	111(52.38)	91(47.42)
	Primary	1041(25.3)	767(23.1)	38(38.38)	23(31.50)
	Secondary and above	545(13.2)	324(9.7)	23(43.80)	8(25.20)
Mother Currently working	Yes	936(22.7)	738(22.2)	42(47.79)	29(41.25)
	No	3184(77.3)	2584(77.8)	140(46.45)	101(41.12)
Family size	> 4	2954(71.7)	2280(68.6)	108(38.37)	78(35.62)
	≤ 4	1166(28.3)	1042(31.4)	74(68.64)	52(53.66)
Wealth index	Rich	1013(24.6)	953(28.7)	20(20.47)	21(22.70)
	Medium	665(16.1)	665(20.0)	22(34.59)	20(31.84)
	Poor	2442(59.3)	1704(51.3)	140(61.40)	89(55.42)
Media exposure	Yes	1441(35.0)	1114(33.5)	24(17.30)	24(22.39)
	No	2679(65.0)	2208(66.5)	158(63.05)	106(50.79)
Improved water source	Yes	2253(54.7)	1814(54.6)	88(40.93)	72(41.43)
	No	1867(45.3)	1508(45.4)	94(53.93)	58(41.00)
Improved toilet facility	Yes	2281(55.4)	1807(54.4)	103(47.91)	68(39.72)
	No	1839(44.6)	1515(45.6)	79(45.32)	62(42.84)
Times to reach water source	< 30 min	1686(40.9)	1583(47.6)	67(42.00)	59(38.74)
	≥ 30 min	2434(59.1)	1739(52.4)	115(50.00)	71(43.39)

Variables measured at the time of the survey: residence, education status, wealth index, sex of household, working status, family size, media exposure, water source, and toilet facility

Variables measured at the time of birth: maternal age (years)

*PMR* perinatal mortality rate

### Reproductive characteristics and perinatal mortality rates

Out of the total 3322 **(**weighted) pregnancies, about 1604(48.3%) of pregnancies were to women who first gave birth when they were < 18 years. The Perinatal mortality among pregnancies was higher in mothers who have a previous history of terminated pregnancy (126.40 per 1000), mothers who have home delivery (52.00 per 1000), mothers who didn’t have participation in making health care decisions (45.38 per 1000), mothers who have a birth interval of < 2 years (99.12 per 1000), mothers who had ≥ 6 parity (86.62 per 1000), mothers who didn’t have ANC follow up (57.78 per 1000), and mothers who have a big problem of distance from the health facility (75.77 per 1000) (Table 2).

**Table 2:** Show that the perinatal mortality among pregnancy by reproductive health characteristics of study participants in high mortality regions, Ethiopia, 2016

Variable	Categories	Frequency (%)	weighted Frequency (%)	unweighted Number of perinatal deaths (PMR [per 1,000])	weighted number of perinatal deaths (PMR [per 1,000])
Age at first birth	< 18 years	2002(48.6)	1604(48.3)	95(50.29)	76(46.62)
	≥ 18 years	2118(51.4)	1718(51.7)	87(43.41)	54(35.32)
Place of delivery	Health institution	1853(45.0)	1453(43.7)	53(29.79)	38(27.35)
	Home	2267(55.0)	1869(56.3)	129(61.02)	92(52.00)
Preceding birth interval (N:2684)	≥ 2 years	2404(72.1)	2183(81.3)	68(29.42)	65(30.98)
	< 2 years	929(27.9)	501(18.7)	75(88.86)	45(99.12)
Women participating in making health care decisions	Yes	3146(76.4)	2881(86.7)	101(33.73)	100(40.00)
	No	974(23.6)	441(13.3)	81(91.11)	30(45.38)
Parity	1–3	1778(43.2)	1533(46.1)	70(41.20)	42(28.59)
	4–6	1435(34.8)	1076(32.4)	59(43.51)	31(30.03)
	≥ 6	907(22.0)	713(21.5)	53(63.25)	57(86.62)
Time to breastfeeding Initiations	Within1hour	1933(46.9)	1637(49.3)	43(22.76)	29(18.57)
	> 1 h	2187(53.1)	1685(50.7)	139(69.36)	101(65.04)
Postnatal care with 2 days	Yes	2618(63.5)	1957(58.9)	107(43.39)	68(36.67)
	No	1502(36.5)	1365(41.1)	75(52.56)	62(47.54)
Ever had a terminated pregnancy	No	3660(88.8)	2949(88.8)	129(37.34)	85(30.32)
	Yes	460(11.2)	373(11.2)	53(120.72)	45(126.40)
Antenatal care visits (N:2456)	≥ 4 visits	952(34.6)	892(36.3)	23(24.46)	25(28.67)
	1–3 visits	934(33.9)	850(34.6)	22(24.10)	20(24.09)
	No at all	868(31.5)	714(29.1)	64(79.11)	39(57.78)
Tetanus toxoid vaccination	Not all	1241 (45.1)	1107(45.1)	64(54.10)	43(40.49)
	One times	371 (13.5)	386(15.7)	7(19.61)	14(37.83)
	≥ 2 +	1141( 41.4)	962(39.2)	37(33.21)	26(27.51)
Distance from Health facilities	Not big problem	2819(68.4)	2315(69.7)	79(29.32)	59(26.55)
	Big problem	1301(31.6)	1007(30.3)	103(85.90)	71(75.770
Health insurance	Yes	3884(94.3)	3018(90.8)	8(34.33)	12(40.00))
	No	236(5.7)	304(9.2)	174(47.54)	118(41.22)

Variables measured at the time of birth: age at first birth, place of delivery, birth interval, parity, time to breastfeeding initiations, postnatal care, antenatal care visits, tetanus toxoid vaccination, and history of terminated pregnancy

Variables measured at the time of the survey: distance from health facilities, health insurance, and women participation in making health care decisions

*PMR* perinatal mortality rate

### Model comparison

The Multicollinearity of the independent variables was checked by using the variation inflation factor (VIF). The VIF was found to range from 1.1 to 1.5 with a mean VIF of 1. 2. This shows that multicollinearity might not be a problem. The DIC values for each model were compared (i.e. model I:1484.8, model II:568.6, model III: 1474.9, and model IV: 566.6) and the model with the lowest value of DIC was selected as a better explanatory model. Based on this, model four with a DIC value (566.6) was selected as the best-fitted model to explain perinatal mortality in high mortality regions of Ethiopia.

### Factors associated with perinatal mortality

In the bivariable multilevel logistic regression analysis, variables like maternal age (years), maternal education status, paternal education status, Sex of household, working status of mothers, age at first birth, health insurance, water facility, and toilet facility had p-value > 0.25 and they were not eligible for multivariable multilevel analysis. In multivariable multilevel analysis, birth interval, media exposure, ANC follow up, time to breastfeeding initiation, maternal parity, previous history of terminated pregnancy, family size, women participation in making health care decisions, and distance from a health facility was significantly associated with perinatal mortality at P-value of ≤ 0.05. Based on this, The odds of perinatal mortality were 3.71 times higher (AOR = 3.71, 95%CI: 2.27, 6.07) among mothers who have < 2 years’ birth interval as compared to mothers who have ≥ 2 years birth interval. The odds of perinatal mortality were 2.43 times higher (AOR = 2.43, 95%CI:1.15,5.38) among mothers who didn’t have ANC visits as compared to mothers who have ≥ 4 visits. The odd of perinatal mortality were 4.01 times higher (AOR = 4.01, 95%CI: 2.49, 6.51) among mothers who didn’t start breastfeeding within one hour of birth as compared to mothers who starts within the first one hour. The odd of perinatal mortality were nearly two times higher (AOR = 1.99, 95%CI: 1.24, 3.22) among mothers who are far from health institutions as compared to their counterparts. The odd of perinatal mortality was 4.68 times higher (AOR = 4.68, 95%CI: 2.76, 7.86) among mothers who have a previous history of terminated pregnancy as compared to mothers who didn’t have a previous history of terminated pregnancy. The odd of perinatal mortality were nearly two times higher (AOR = 1.96, 95%CI:1.19, 3.20) among mothers who didn’t have autonomy in making health care decisions as compared to their counterparts. The odd of perinatal mortality among mothers who didn’t have media exposure was 2.78 times higher (AOR = 2.78, 95%CI:1.48, 5.59) as compared to mothers who have media exposure. Being Households ≤ 4 family sizes were associated with a higher odds of perinatal mortality (AOR = 4.12, 95%CI:2.19, 7.79) as compared to households with greater than four family sizes. The odd of perinatal mortality among mothers who have ≥ 6 parity were 2.48 times higher (AOR = 2.48, 95%CI:1.21,5.22) as compared to mothers who have one to three parity (Table 3).

**Table 3:** Shows the multivariable multilevel analysis of factors associated with perinatal mortality in high mortality regions, Ethiopia, 2016

Variable		Null	Model II AOR (95%CI)	Model III AOR (95%CI)	Model IV AOR (95%CI)
Wealth index	Rich		1		1
	Medium		1.45( 0.61, 3.48)		1.37( 0.58,3.32)
	Poor		1.06( 0.53, 2.27)		0.92(0.45,2.00)
Birth interval in years	≥ 2 years		1		1
	< 2 years		3.72( 2.28, 6.0)*		3.71(2.27,6.07)*
Media exposure	Exposed		1		1
	Not exposed		2.79( 1.49, 5.60)*		2.78(1.48, 5.59)*
Antenatal care visits	≥ 4 visits		1		1
	1–3 visits		0.82( 0.39, 1.73)		0.79(0.37,1.69)
	No at all		2.51( 1.18,5.54)*		2.43(1.15,5.38)*
Tetanus toxoid vaccination	Not all		1		1
	1		0.59(0 .22,1.38)		0.56(0.21,1.33)
	≥ 2 +		0.68( 0.36, 1.29)		0.68(0.36,1.29)
Place of delivery	Health Institution		1		1
	Home		1.23( 0.71, 2.16)		1.25(0.72,2.22)
Time to breastfeeding initiation	With 1 h		1		1
	> 1 h		3.95( 2.50, 6.40)		4.01(2.49,6.51)*
Post natal follow up with in 2 day	Yes		1		1
	No		0.93( 0.53, 1.66)		0.93( 0.53,1.67)
Parity	1–3		1		1
	4–6		1.53(0.78,3.03)		1.54(0.79,3.08)
	≥ 6		2.55(1.24,5.37)*		2.48(1.21,5.22)*
Ever had terminated pregnancy	No		1		1
	Yes		4.68(2.76,7.86)*		4.68(2.75, 7.86)*
Family size	> 4		1		1
	≤ 4		4.06(2.15,7.69)*		4.12(2.19,7.79)*
Women participating in making health care decisions	Yes		1		
	No		1.97(1.20,3.21)*		1.96(1.19, 3.20)*
Distance from health facility	Not big problem			1	1
	Big problem			2.80(2.06,3.82)*	1.99(1.24, 3.22)*
Residence	Urban			1	1
	Rural			1.53 (0.95, 2.61)	0.93 (0.44, 2.19)
Time to reach to a water source	< 30 min			1	1
	≥ 30 min			1.32(0.97, 1.80)	1.38 ( 0.85, 2.30)

1: Reference *OR* Adjusted Odds Ratio, *CI* Confidence Interval

^*^ = *P* ≤ 0.05

## Discussion

The study aimed to identify the individual and community-level factors of perinatal mortality in high mortality regions of Ethiopia. Our study found that birth interval, media exposure, ANC follow up, time to breastfeeding initiation, maternal parity, previous history of terminated pregnancy, family size, women participation health care decisions and distance from a health facility were significant predictors of perinatal mortality.

The study revealed that mothers who gave birth in less than 2-year intervals have higher odds of perinatal mortality as compared to those who gave birth after 2-year intervals. The finding is supported by other studies [12, 19, 20, 25, 26, 28]. The possible explanation may be due to that short birth intervals hurt the nutritional recovery, healing of the endometrium, cervical competency, and the optimal lactation of the breast which determines the outcomes of the subsequent pregnancy [29]. Thus, measures should be taken to promote family planning utilization to space the succeeding pregnancy.

The odds of perinatal mortality were higher among mothers who didn’t have antenatal care visits as compared to mothers who have four and above antenatal care visits. The finding is supported by other studies [7, 14–16, 19]. The possible justification might be that having more antenatal care visits was an opportunity for health professionals to give health education, identify pregnancy with complications, and treat an existing disease [30]. In addition, having a quality antenatal care service will facilitate mothers to deliver in a health institution, and increase mothers’ knowledge regarding the care that has been given in health institutions such as essential newborn care [31].

The study revealed that the mothers who have no autonomy in making health care decisions are more likely to have higher odds of perinatal mortality as compared to their counterparts. This is in agreement with the study conducted in Bangladesh [20]. The possible explanation may be due to those women who are autonomous in making health care decisions were more likely to have antenatal follow up [32–34], postnatal care [32, 34], and delivered at the health facility [33, 34]. It is crucial to identify and treat an existing complication in pregnancy and early in the newborn baby.

Unlike the study done in Bangladesh [20], in this study, mothers who have no media exposure have higher odds of perinatal mortality as compared to their counterparts. The possible justifications for such findings may be due to that those mothers who have exposure to media are in advantage of getting information regarding preventive measures of perinatal mortality such as maternal and child health services utilization. In addition, they might have information on complications related to pregnancy and childbirth which urges the mother for seeking early health care.

In agreement with studies [14, 25], this study found that mothers who have a previous history of terminated pregnancy are associated with a high odds of perinatal mortality as compared to their counterparts. This might be due that the genetic make-up of mothers and repeated exposure to environmental factors or this might be due to the problems related to the reproductive organs such as the uterus, and cervix or it may be due to problems related to the anatomical structure of the pelvic floor. In addition to this, it can be explained as the cultural impact in the community may force the couples to replace the lost fetus or newborn within a short birth interval without full recovery of mothers; this will determine the outcomes of pregnancy. Thus, mothers should be linked to reproductive health services and counseled on birth intervals. In addition, special investigation and care should be considered in the subsequent pregnancy.

Late initiation of breastfeeding (after one hour) is another factor that shows high odds of perinatal mortality (early newborn). The finding is supported by the study conducted in India [35]. The possible reason might be that timely initiation of breastfeeding is an assurance that a newborn has received colostrum; which contains infection protecting antibodies and reduces early newborn mortality resulting from infectious diseases. Thus, measures such as health education in every birth are needed to initiate breastfeeding within one hour of birth.

Unlink to the other study [36], this study found that being far from the health institutions is significantly associated with higher odds of perinatal mortality. The proximity to a health institution will reduce the burden of transportation and help the mother have antenatal care and institutional delivery, which was an important opportunity to screen and treat an existing disease in early newborn and pregnant mothers. This has a programmatic implication that the maternal waiting service should be strengthened and special consideration should be given to pregnant mothers thus far from health institutions.

Being Households ≤ 4 family sizes were associated with higher odds of perinatal mortality as compared to households who had greater than four family sizes. The finding is supported by a study in West Gojam zone [22]. The possible explanation might be that the large family members will reduce the workload of pregnant mothers in homes and give enough time for resting and attending hospital follow-up care such as antenatal and postnatal care. Such care will have an advantage on the outcomes of the pregnancy.

The odd of perinatal mortality is higher among mother who has six parity compared to mothers who have one to three parity. The finding is in agreement with the study conducted in Sekota [15] and contrary to the study has done in Jimma [14]. This may be due to the reason that the repeated pregnancy in grand multipara mothers may lead to fetal nutritional deprivation which can cause either stillbirth or early neonatal death. Thus, grand Para mothers should be seen in special during antenatal care visits.

In the Previous study, lower socioeconomic status was reported as contributing factor of adverse pregnancy outcomes [37], on the contrary, this study revealed that the wealth index was not independently associated with perinatal mortality after controlling for other individuals and community-level characteristics. This might be due to the free provision of the preventive measure of perinatal mortality such as maternal and child health services in Ethiopia to all mothers irrespective of their economic status.

Whereas prior studies have linked maternal education status to perinatal mortality [17, 18], our findings are similar to previous studies in Ethiopia [14, 15, 19, 22, 23, 26, 27] found no association between perinatal death and maternal education status. The possible justification might be due to the home-to-home service of health extension workers in Ethiopia to all mothers regardless of the educational status, such as the provision of health education, screening mothers with obstetrics complications, and early referring including newborns with complications.

The clinical and public health implication of this study is to take prompt intervention on the determinant factors and response to halt the high burden of perinatal mortality. Therefore, considering and taking special attention to birth spacing, antenatal care utilization, time to breastfeeding initiation of a newborn, the mothers who are distant from health institutions, the mothers have a previous history of a terminated pregnancy, empowering mother participation in making health care decision, mothers have no exposure to media, mothers from large household size and the Grand Para mothers could reduce perinatal mortality in high mortality regions of Ethiopia.

### Strength and limitation

The study has used nationality representative data of 2016 EDHS and which is considered to have good generalizability. Besides, we have employed an appropriate statistical approach (multilevel mixed analysis) to estimate the cluster effect on perinatal mortality. However, the study shares the limitation of a cross-sectional study that was impossible to establish the cause and effect relationship. This study might have a recall bias, particularly for variables such as age, preceding birth interval, parity, time to breastfeeding initiation, postnatal care, and antenatal care visits or other retrospective data relying on memory of past events. Inaccurate reporting of respondents on crucial variables (gestational age, birth dates, and ages at death) used for estimating perinatal mortality, may result in underestimation or overestimation of perinatal mortality. The study is not considered the recommended birth weight of fetuses (≥ 1000gm) to estimate the stillbirth of perinatal mortality, hence, we only used the gestational age of fetuses; fetuses blew this weight may be incorporated in the study and may lead to overestimation of the mortality. This study has only analyzed the outcomes of the last pregnancy. Thus, the pregnancies to women with higher parity and shorter birth intervals might be excluded. This might lower the perinatal mortality and have an impact on the association of parity, birth intervals with perinatal mortality. The other limitation of this study is raised from the missing data that the pooled result of the perinatal mortality rate was lowered due to the pregnancies that had higher perinatal mortality being excluded from the analysis.

## Conclusion

The study concludes that birth interval, antenatal care, time to breastfeeding initiation, distance from health institutions and previous history of a terminated pregnancy, maternal autonomy, media exposure, family size, and parity were predictors of prenatal mortality. Therefore, programmatic emphases to maternal waiting service utilization for mothers distant from health institutions and media advertising regarding the complications related to pregnancy, childbirth and on its respective direction that the mothers should follow could reduce perinatal mortality in high mortality regions of Ethiopia**.** In addition**,** strengthening maternal and child health services utilization and empowering mothers in making health care decisions should be emphasized.

## Data Availability

The data is available at the corresponding Author and may be provided upon request.

## Appendix

Comparisons of perinatal death between the final Analytic Sample with the Excluded Samples Due to Missing data

**Table:**

Variable	Category	Analytic sample	Missing sample	X^2^ -test	*P*-value
		Weighted frequency (%)	Weighted frequency (%)		
Perinatal death	Yes	130(3.9)	17( 4.4)	0.414	0.255
	No	3192(96.1)	359( 95.6)		

## Abbreviations

ANC: Antenatal care
AOR: Adjusted odds ratio
CI: Confidence intervals
CSA: Central Statistical Agency
DIC: Deviance information criterion
EAs: Enumeration areas
EDHS: Ethiopian Demographic and Health Survey
TT: Tetanus toxoid
ENAP: Every Newborn Action Plan
